# SIRT2 and NAD ^+^ Boosting Broadly Suppress Aging‐Associated Inflammation

**DOI:** 10.1111/acel.70162

**Published:** 2025-07-04

**Authors:** Marine Barthez, Zehan Song, Yufan Feng, Yifei Wang, Chih‐ling Wang, Danica Chen

**Affiliations:** ^1^ Department of Nutritional Sciences and Toxicology University of California Berkeley California USA; ^2^ Metabolic Biology Graduate Program University of California Berkeley California USA

## Abstract

Aging leads to chronic inflammation that is linked to aging‐associated conditions and diseases. Multiple immune pathways become activated during aging, posing a challenge to effectively reduce aging‐associated inflammation. SIRT2, an NAD^+^‐dependent deacetylase, suppresses several immune pathways that become activated during aging and may represent an attractive target to broadly dampen aging‐associated inflammation. Here, we show that SIRT2 deficiency leads to increased inflammation governed by multiple immune pathways and tissue function decline at an old age, while NAD^+^ boosting with 78c suppresses aging‐associated inflammation and improves tissue function. These findings highlight SIRT2 as a master regulator of aging‐associated inflammation and support NAD^+^ boosting as an effective strategy to counteract aging‐associated inflammation and tissue function decline.

## Graphical abstract

Barthez et al. show that SIRT2 deficiency amplifies aging‐associated inflammation and tissue dysfunction via multiple immune pathways. NAD+ boosting suppresses inflammation and restores tissue function. This study underscores the central role of SIRT2 in regulating aging‐associated inflammation.

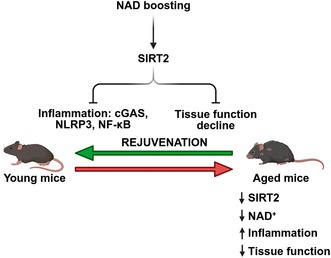

## 1 Introduction

Aging is a complex process that induces changes at the molecular, cellular, and organismal levels, ultimately contributing to the onset of aging‐related conditions and diseases. One hallmark of aging is chronic low‐grade inflammation, commonly referred to as “inflammaging” (López‐Otín et al. 2023). Several immune pathways have been shown to be activated during aging, contributing to aging‐associated inflammation. Nuclear Factor Kappa B (NF‐κB), a key transcription factor that drives the expression of pro‐inflammatory cytokines such as TNF‐α and IL‐6, is activated during aging (G. Zhang et al. 2013). The NLRP3 inflammasome is activated during aging, leading to increased production of inflammatory cytokines IL‐1β and IL‐18 (He et al. 2020). The cGAS‐STING pathway responsible for immune sensing of cytosolic DNA also becomes activated with aging, leading to sustained type I interferon response (De Cecco et al. 2019; Simon et al. 2019). These observations suggest the complexity of aging‐associated inflammation and the challenge to effectively dampen aging‐associated inflammation.

While these immune pathways were thought to be activated during aging due to the accumulation of damage‐associated molecular patterns (DAMPs), recent studies suggest that these immune pathways become aberrantly regulated with aging, resulting in a reduced ability to suppress inflammation and return to homeostasis. Prominently, SIRT2, an NAD^+^‐dependent deacetylase, simultaneously suppresses multiple immune pathways that are activated during aging. By deacetylating the p65 subunit of NF‐κB, SIRT2 reduces its nuclear translocation and suppresses pro‐inflammatory gene expression (Rothgiesser et al. 2010; Yuan et al. 2016). Acetylation of NLRP3 facilitates the assembly of the NLRP3 inflammasome and the production of IL‐1β and IL‐18, while deacetylation of NLRP3 by SIRT2 prevents the assembly of the NLRP3 inflammasome and the production of the cytokines (Luo et al. 2019; He et al. 2020). Furthermore, cGAS is also decorated by acetylation. SIRT2 deacetylates cGAS and suppresses cGAS‐STING signaling and the type I interferon response (Barthez et al. 2025). Finally, SIRT2 deacetylates STAT3 and suppresses STAT3 signaling, which is activated by pro‐inflammatory cytokines (Ye et al. 2023). During aging, the expression of SIRT2 is reduced (Luo et al. 2019; He et al. 2020; Barthez et al. 2025). These observations suggest that SIRT2 may represent an attractive target to broadly dampen aging‐associated inflammation.

Aging is marked by a decline in NAD^+^ levels, contributing to metabolic dysfunction and increased susceptibility to age‐related diseases. A key driver of this decline is the age‐associated upregulation of CD38, a major NAD^+^‐consuming enzyme. 78c is a well‐characterized, potent CD38 inhibitor that restores NAD^+^ levels and has been shown to extend lifespan and improve physiological function in aged mice (Peclat et al. 2022; Tarragó et al. 2018).

In this study, we tested this hypothesis by characterizing both aged SIRT2 knockout (KO) mice and aged wild type (WT) mice treated with an NAD^+^ booster 78c to activate SIRT2 for inflammation markers and tissue function. We found that aged SIRT2 KO mice showed increased broad inflammation markers and compromised tissue function, while aged WT mice treated with 78c showed reduced inflammation and improved tissue function. Our study supports SIRT2 as a master regulator of aging‐associated inflammation and NAD^+^ boosting as an effective strategy to dampen aging‐associated inflammation.

## 2 Results

### 2.1 SIRT2 Suppresses Aging‐Associated Inflammation and Tissue Function Decline

**FIGURE 1:**
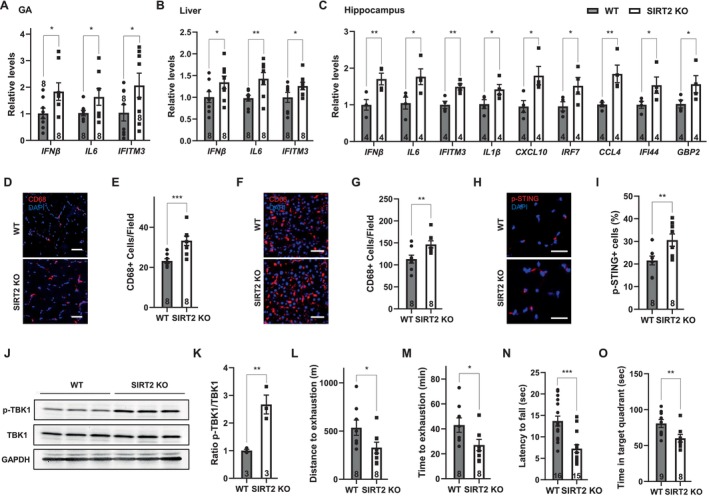
SIRT2 suppresses aging‐associated inflammation and tissue function decline. Comparison of aged (12–25 months old) WT and SIRT2 KO mice. (A–C), Quantitative real‐time PCR analyses for the mRNA levels of the indicated genes in the GA (A, *n* = 8), the liver (B, *n* = 8), and the hippocampus (C, *n* = 4). (D–G), Immunostaining for CD68+ cells and quantification of the GA (D, E) and the liver (F, G) sections. Scale bar: 50 μm. *n* = 8, 5 images examined from 3 slides/mouse. (H, I), Immunostaining for phosphorylated STING and quantification of the GA sections. Scale bar: 30 μm. *n* = 8, 4 images examined from 3 slides/mouse. (J, K), Western blotting analyses and quantification of TBK1 and phosphorylated TBK1 in the liver. GAPDH was used as a control. *n* = 3. (L, M), Distance and time to exhaustion in the treadmill exhaustion test. *n* = 8. (N), Latency to fall in the grip strength test. *n* = 16, 15. (O), Time spent in the target quadrant in the Barnes maze test. *n* = 9, 8. Data are mean ± s.e.m. *p < 0.05. **p < 0.01. ***p < 0.001. Student's *t* test.

SIRT2 KO mice do not exhibit noticeable phenotypes at a young age (He et al. 2020). We characterized aged SIRT2 KO mice and their WT controls for inflammation markers and tissue function. The expression of inflammatory cytokines and interferon‐stimulated genes (ISGs) was increased in the tissues of aged SIRT2 KO mice (Figure 1A–C). Macrophage infiltration, indicated by CD68‐positive cells, was increased in the tissues of aged SIRT2 KO mice (Figure 1D–G). cGAS‐STING signaling became aberrantly upregulated in the tissues of aged SIRT2 KO mice, as demonstrated by increased phosphorylation of STING (Figure 1H,I) and increased phosphorylation of TBK1 (Figure 1J,K), a kinase downstream of cGAS signaling (Zhao et al. 2019; C. Zhang et al. 2019). Inflammatory cytokines, such as TNF‐α, IL‐6, and IL‐18, were increased in the serum of aged SIRT2 KO mice (He et al. 2020; Barthez et al. 2025). Interestingly, while inflammation was increased in the hippocampus of aged SIRT2 KO mice, it was unchanged in the cortex (Figure S1). This is consistent with the observations that aging‐related increases in inflammatory cytokines are more pronounced in the hippocampus compared to the cortex, either due to increased density of microglia or more pronounced aging‐related breakdown of the blood–brain barrier in the hippocampus (Porcher et al. 2021; Biscetti et al. 2025; Lawson et al. 1990; Montagne et al. 2015). Together, these data suggest SIRT2 broadly suppresses aging‐associated inflammation.

Aged SIRT2 KO mice showed impaired tissue function, including compromised muscle function, as evidenced by reduced distance and time to exhaustion in the treadmill exhaustion test (Figure 1L,M) and decreased latency to fall in the grip strength test (Figure 1N). Cognitive decline was also apparent, as aged SIRT2 KO mice spent less time in the target quadrant in the Barnes maze test (Figure 1O). In contrast, SIRT2 KO mice performed competently in the open field test and the elevated plus maze test (Figure S2A–D). SIRT2 KO mice also displayed dysregulated metabolic function, including increased fat mass (Figure S2E–H) and glucose intolerance (He et al. 2020). Together, these data indicate that SIRT2 suppresses aging‐associated inflammation and tissue function decline.

### 2.2 78c Suppresses Aging‐Associated Inflammation and Tissue Function Decline

**FIGURE 2:**
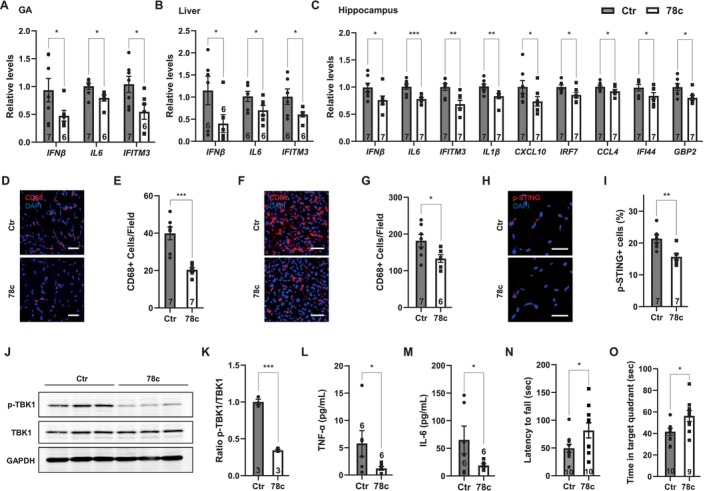
78c suppresses aging‐associated inflammation and tissue function decline. Comparison of aged (24 months old) WT mice treated with or without 78c for 2 months. (A–C), Quantitative real‐time PCR analyses for the mRNA levels of the indicated genes in the GA (A, *n* = 7,6), the liver (B, *n* = 6) and the hippocampus (C, *n* = 7). (D–G), Immunostaining for CD68+ cells and quantification of the GA (D, E, *n* = 7) and the liver (F, G, *n* = 7,6) sections. Scale bar: 50 μm. 5 images examined from 3 slides/mouse. (H, I), Immunostaining for phosphorylated STING and quantification of the GA sections. Scale bar: 30 μm. *n* = 7.4 images examined from 3 slides/mouse. (J, K), Western blotting analyses and quantification of TBK1 and phosphorylated TBK1 in the liver. GAPDH was used as a control. *n* = 3. (L, M), Levels of TNF‐α and IL‐6 in the serum. *n* = 6. (N), Latency to fall in the grip strength test. *n* = 10. (O), Time spent in the target quadrant in the Barnes maze test. *n* = 10, 9. Data are mean ± s.e.m. *p < 0.05. **p < 0.01. ***p < 0.001. Student's *t* test.

We next tested whether NAD^+^ boosting is sufficient to activate SIRT2 and suppress aging‐associated inflammation and tissue function decline. We treated 24‐month‐old WT mice with 78c, an NAD^+^ booster (Peclat et al. 2022; Haffner et al. 2015), for 2 months. 78c treatment reduced the expression of inflammatory cytokines and ISGs in the tissues (Figure 2A–C), reduced macrophage infiltration in the tissues (Figure 2D–G), decreased cGAS‐STING signaling (Figure 2H–K), reduced inflammatory cytokines in the serum (Figure 2L,M), and improved muscle function (Figure 2N) and cognitive function (Figure 2O). Collectively, these data suggest that NAD^+^ boosting via 78c enhances SIRT2 function, thereby suppressing aging‐associated inflammation and tissue function decline.

## 3 Discussion

Our findings support SIRT2 as a master regulator of aging‐associated inflammation. Aged SIRT2 KO mice show increased levels of inflammatory cytokines that are regulated by multiple aging‐associated immune pathways, including NF‐κB, the NLRP3 inflammasome, and the cGAS‐STING signaling, consistent with the reports that SIRT2 regulates the key components of these immune pathways through deacetylation (Figure 1) (He et al. 2020; Barthez et al. 2025; Rothgiesser et al. 2010; Ye et al. 2023; Yuan et al. 2016). Aged SIRT2 KO mice exhibit compromised muscle function, cognitive function, stem cell exhaustion, metabolic disorder, and susceptibility to severe COVID‐19 (Figures 1,S2) (He et al. 2020; Barthez et al. 2025; Luo et al. 2019; Tang et al. 2017; Y. Zhang et al. 2023; Fourcade et al. 2017; Ma et al. 2022), consistent with the notion that aging‐associated inflammation is linked to aging‐associated conditions and diseases.

Our study supports NAD^+^ boosting as an effective strategy to suppress aging‐associated inflammation, contributing to lifespan and health span extension. Aged WT mice treated with 78c show reduced levels of inflammatory cytokines, consistent with SIRT2 activation (Figure 2). It is important to note that CD38 inhibition by 78c increases NAD^+^ levels systemically and is likely to impact other sirtuins and NAD^+^‐dependent pathways. Thus, the observed benefits may not be exclusively mediated by SIRT2. In accord with reduced inflammation, 78c treatment improves muscle function, cognitive function, stem cell maintenance, and metabolic homeostasis, alleviates fatty liver and severe COVID‐19, and extends lifespan (Figure 2) (Song et al. 2024; Barthez et al. 2025; Ohkubo et al. 2022; Tarragó et al. 2018; Peclat et al. 2022).

## 4 Materials and Methods

### 4.1 Mice

SIRT2 KO mice have been described previously (Luo et al. 2019; He et al. 2020). For experiments using WT mice, C57BL/6 mice were obtained from the National Institute on Aging. 78c (MedChemExpress LLC, #HY‐123999) was administered to mice by intraperitoneal injection (10 mg/kg/dose) twice daily. Control mice received vehicle (5% DMSO, 15% PEG400, 80% of 15% hydroxypropyl‐g‐cyclodextrin (in citrate buffer pH 6.0)) injections. Mice were housed on a 12:12 h light: dark cycle at 25°C with ad libitum access to water and standard laboratory chow diet provided by LabDiet (0007688). All animal procedures were in accordance with the Animal Care Committee at the University of California, Berkeley.

### 4.2 Quantitative Real‐Time PCR

Total RNA was isolated from tissue homogenates using Trizol reagent (Invitrogen), converted to complementary DNA using the qScript cDNA SuperMix (Quanta Biosciences) and gene expression was determined by real‐time PCR using the Eva qPCR SuperMix kit (BioChain Institute) on an ABI StepOnePlus system. The primer sequences are listed in Table S1. The 2^−ΔΔCt^ method was used to analyze gene expression fold change after normalization with GAPDH and then to the controls.

### 4.3 Immunofluorescence Microscopy

OCT‐embedded liver and GA were sectioned at a thickness of 10 μm. Sections were fixed with 4% formaldehyde for 1 h, washed with PBS, and incubated for 1 h in blocking solution (0.3% H_2_O_2_, 0.2% Triton X‐100, 2% goat serum in PBS). The slides were incubated with anti‐CD68 antibody (1:200, BioLegend, 137,001) or anti‐p‐STING antibody (1:200, Cell Signaling, #62912S) overnight at 4°C. After washes, the sections were incubated with secondary antibodies for 1 h. Sections were imaged on a Zeiss LSM 880 FCS inverted confocal microscope. The positive cells were manually counted or counted using Image J.

### 4.4 Western Blot

Tissues were homogenized in a lysis buffer that contained protease and phosphatase inhibitors. Supernatants were collected, and total protein was quantified with Pierce Bradford Plus Protein Assay Kits (Thermo Scientific, 23236). Proteins were resolved by SDS‐PAGE and transferred to nitrocellulose membranes (Bio‐Rad), which were incubated with antibodies for TBK1 (CST, 3504), phospho‐TBK1 (CST, 5483) and GAPDH (CST, 5174). Membranes were then washed and incubated for 60 min with 1:2000 HRP‐conjugated secondary antibody (BioLegend). After further washes, the membranes were exposed to enhanced chemiluminescence substrate (PerkinElmer, NEL103001EA) and visualized using ImageQuant LAS 4000 (GE Healthcare).

### 4.5 Grip Strength Test

As described (Ueno et al. 2022), mice were habituated in the procedural room for 5–20 min. Mice were put in the center of the wire cage top, inverted, and suspended 40–50 cm above a padded surface. The latency until mice fell was recorded. The test was repeated three times for each mouse to calculate average latency.

### 4.6 Treadmill Exhaustion Test

As described (Damal Villivalam et al. 2021), mice were acclimated to the treadmill (Columbus Instruments, Exer‐6 M Open Treadmill) 2–3 days prior to the test. For each session, food was removed 2 h before exercise. Mice were familiarized with the sound and experience of moving treadmill for two training sessions (1 session per day). During the training session, mice were put on the stationary treadmill for 30 s to explore the environment. Acclimation began at a low speed of 5–8 m per minute (m/min) for 10 min on Day 1 and was increased to 5–10 m/min for 10 min on Day 2. Following training, mice were allowed to rest for 1 day in their home cage before the exhaustion test.

The treadmill exhaustion test began at a rate of 12 m/min for the first 40 min. After 40 min, treadmill speed was increased at a rate of 1 m/min every 10 min for 30 min, and then was increased at a rate of 1 m/min every 5 min until mice were exhausted. Exhaustion was determined by failure to remain on the treadmill for at least 20 s. Time and distance to exhaustion were recorded.

### 4.7 Barnes Maze Test

Mice were trained on the Barnes maze as previously described (Wang et al. 2023). The maze was a circular platform made of acrylic plastic (92 cm in diameter) with 20 identical holes (5 cm in diameter), one of which was the target hole with an escape box placed underneath it. The test included three phases: habituation (Day 1), training trials (Days 2–3), and probe trials (Day 5). Mice were acclimated to the procedural room for 1 h every day before the session.

For habituation, the mouse was placed at the center of the maze under a clear beaker for 30 s while the noise was displayed via a metronome (65 dB, 1 beat/s). The mouse was slowly guided straight to the target hole using the clear beaker and was given 3 min to enter the escape box through the target hole. If the mouse failed to escape within 3 min, it was placed inside the escape box. The mouse was allowed to stay in the escape box for 1 min with the noise turned off.

During the training phase, three training trials were performed on Day 1, and two training trials were performed on Day 2. A mouse was placed at the center of the maze under an opaque beaker for 10 s. At the end of the holding period, noise was turned on and the mouse was released from the opaque beaker to explore for 2 min. If the mouse failed to escape at the end of the exploration time, it was guided to the target hole with a clear beaker and was given 3 min to escape. If the mouse failed to escape within 3 min, it was placed inside the escape box. Once the mouse escaped, noise was turned off and it was allowed to stay in the escape box for 1 min.

Probe trials were carried out on Day 5. The setup was the same as training trials, except the escape box was removed from the maze. The mouse was allowed to explore the maze for 2 min with noise turned on. Mouse performance was recorded with a digital camera recorder and videos were analyzed with Noldus EthoVision (Noldus Information Technology, Wageningen, The Netherlands).

### 4.8 Open Field Test

As described previously (Wang et al. 2023), mice were transferred to and allowed to habituate in the testing room under normal light for 60 min before testing. Mice were placed in a clear plastic chamber (50 × 50 cm) for 15 min. Mouse movement was recorded with a digital camera recorder, and the videos were analyzed with Ethvision (Noldus Information Technology, Wageningen, The Netherlands).

### 4.9 Elevated Plus Maze

As described previously (Wang et al. 2023), mice were transferred to and allowed to habituate in the testing room under normal light for 60 min before testing. Mice were allowed to explore an elevated plus maze that consisted of open and closed arms for 10 min. Entries into closed and open arms were recorded with a digital camera recorder, and the videos were analyzed with EthoVision (Noldus Information Technology, Wageningen, The Netherlands).

### 4.10 Mouse Serum MultiPlex Cytokine Measurements

Cytokines in mouse sera were measured with the Luminex Mouse cytokine immunoassay for IL‐6 and TNF‐α (Millipore, MCYTOMAG‐70 K) on a Bio‐Plex MAGPIX Multiplex Reader according to the manufacturer's instructions.

### 4.11 Echo MRI

Mouse body composition (total body fat and lean mass measurements) was measured using an EchoMRI‐100 V Body Composition Analyzer (EchoMRI).

### 4.12 Quantification and Statistical Analysis

The number of biological replicates was chosen based on the nature of the experiments and published papers describing similar experiments. No statistical methods were used to predetermine sample sizes. Sample size (*n*) can be found on the bar graphs representing the biological replicates (the number of mice) or the technical replicates. Mice were randomized to groups, and analysis of mice and tissue samples was performed by investigators blinded to the treatment or the genetic background of the animals. Statistical analysis was performed with Student's *t*‐test (GraphPad Prism software). Data are presented as means, and error bars represent standard errors. In all corresponding figures, * represents *p* < 0.05. ** represents *p* < 0.01. *** represents *p* < 0.001. ns represents *p* > 0.05.

## Author Contributions

D.C. conceived the study. M.B. performed most experiments. Z.S. treated mice with 78c. Y.F., Y.W., and C.W. assisted with characterizations of mice. D.C. wrote the manuscript with the help of M.B. and other authors.

## Conflicts of Interest

The authors declare no conflicts of interest.

## Supporting information


Data S1.

Data S2.

## Data Availability

The data that support the findings of this study are available from the corresponding author upon reasonable request.
