# Supplementary material for: SIRT2 and NAD + Boosting Broadly Suppress Aging‐Associated Inflammation
Source: Aging Cell. 2025 Jul 4;24(9):e70162. doi: 10.1111/acel.70162 (PMC12419836; doi:10.1111/acel.70162)
Supplement: Supplementary file 1 — Data S1. [file ACEL-24-e70162-s002.pdf]

AGING CELL AUTHOR CHECKLIST. ***Authors should submit this checklist together with their manuscript. Please ensure that you have read the Author Guidelines in detail before submission.***

**Title**	SIRT2 and NAD+ boosting broadly suppress aging-associated inflammation
**Authors**	Marine Barthez, Zehan Song, Yufan Feng, Yifei Wang, Chih-ling Wang, Danica Chen
**Manuscript Type**	Short communication
**Total Character Count (including spaces)[1]**	19,817
**Word count of Summary[2]**	114
**Number of papers cited in the References[3]**	28
**Listing of all Tables (Table1, Table 2 etc)[4]**	Supplementary Table 1

**Figure specifications (please complete one row per figure)[5]** ***Figure no.***	Colour ***(yes/no)***	Greyscale ***(yes/no)***	Black and white ***(yes/no)***	Single column (80mm) ***(yes/no)***	Double column (180mm) ***(yes/no)***	Size of figure at full scale (mm x mm) ***(insert details)***	Smallest font size used in the figure at full scale (minimum 6pt) ***(insert details)***
Figure 1	yes	yes	no	no	yes	190.5 x 254	11
Figure 2	yes	yes	no	no	yes	190.5 x 254	11
Figure S1	no	yes	no	no	yes	190.5 x 254	10
Figure S2	no	yes	no	no	yes	190.5 x 254	10

[1] The maximum character count allowed is 50,000 (incl. spaces) for Primary Research Papers and Reviews, 10,000 for Short Takes.

[2] Summary should not exceed 250 words.

[3] Primary Research Papers can contain a maximum of two tables. If more are needed they should replace some of the Figures or can be placed in the Supporting Information.

[4] A maximum of 45 references is allowed for Primary Research Papers and 20 references for Short Takes.

[5] A Primary Research Paper may contain up to 6 figures and a Short Take up to 2 figures. Authors are encouraged to provide figures in the size they are to appear in the journal and at the specifications given.
