# Supplementary material for: SIRT2 and NAD + Boosting Broadly Suppress Aging‐Associated Inflammation
Source: Aging Cell. 2025 Jul 4;24(9):e70162. doi: 10.1111/acel.70162 (PMC12419836; doi:10.1111/acel.70162)
Supplement: Supplementary file 2 — Data S2. [file ACEL-24-e70162-s001.pdf]

Supplementary Material for

SIRT2 and NAD$^+$ boosting broadly suppress aging-associated inflammation

Marine Barthez[1], Zehan Song[1,2], Yufan Feng[1], Yifei Wang[1,2], Chih-ling Wang[1], Danica Chen[1*]

[1]Department of Nutritional Sciences and Toxicology, University of California, Berkeley, CA 94720, USA.
[2]Metabolic Biology Graduate Program, University of California, Berkeley, CA 94720, USA.
[*]Correspondence. danicac@berkeley.edu

This file includes:

Figures S1 to S2
Table S1

Figure S1. SIRT2 regulation of aging-associated inflammation

A

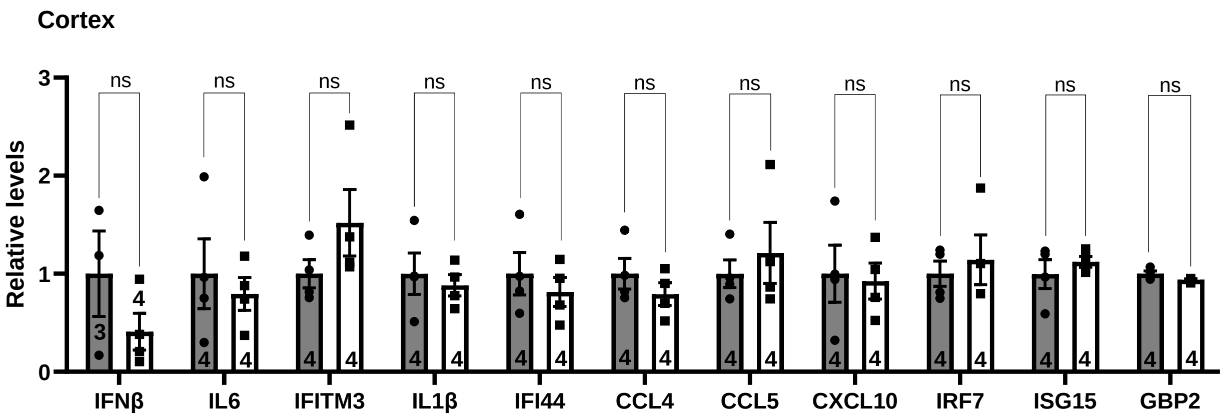

B

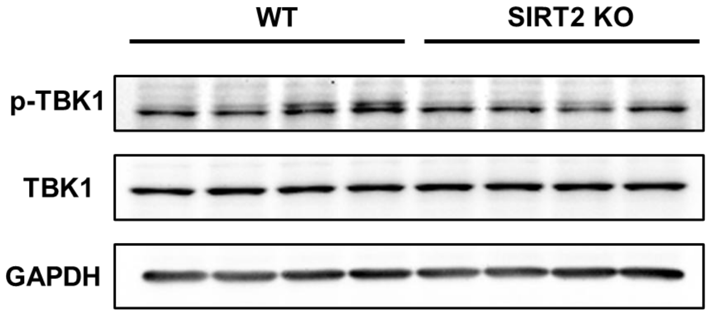

Figure S1. SIRT2 regulation of aging-associated inflammation.

Comparison of aged (24 months old) WT and SIRT2 KO mice.

A, Quantitative real-time PCR analyses for the mRNA levels of the indicated genes in the cortex. n=4.

B, Western blotting analyses of TBK1 and phosphorylated TBK1 in the cortex. GAPDH was used as a control. n=3.

Data are mean ± s.e.m. ns $p>0.05$. Student’s t test.

Figure S2. SIRT2 prevents aging-associated tissue function decline

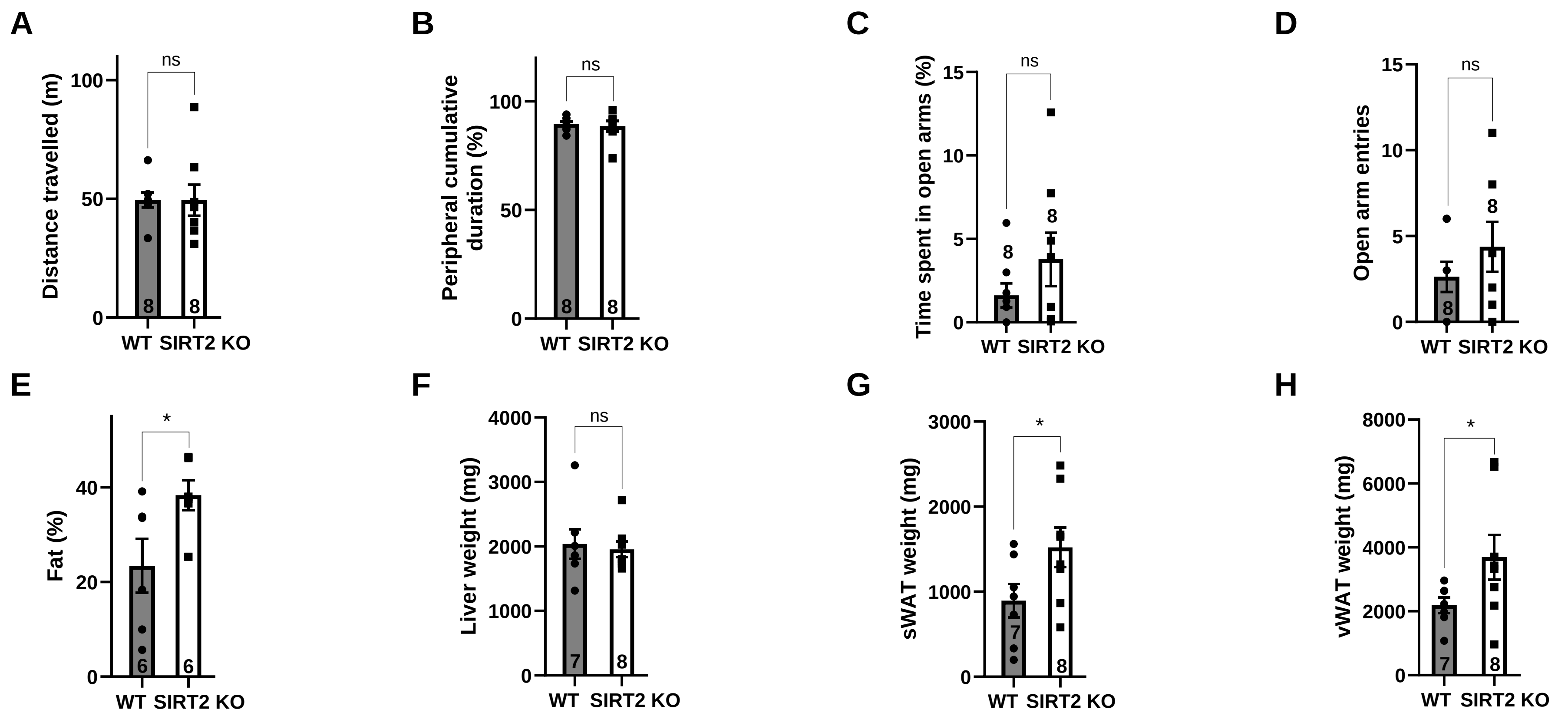

Figure S2. SIRT2 prevents aging-associated tissue function decline.
Comparison of aged (12-24 months old) WT and SIRT2 KO mice.
A,B, Distance travelled and peripheral cumulative duration in the open field test. n=8.
C,D, Time spent in open arms and number of open arm entries in the elevated plus maze test. n=8.
E, Mouse body composition determined by Echo MRI. n=6.
F-H, Tissue weight for the liver (F), sWAT (G), and vWAT (H). n=7,8.
Data are mean ± s.e.m. ns $p>0.05$. * $p < 0.05$. Student’s t test.

Supplementary Table 1. Primers used in qPCR

mGAPDH	Forward	CCC ATC ACC ATC TTC CAG GAG C
	Reverse	CCA GTG AGC TTC CCG TTC AGC
mIFNβ	Forward	GCA CTG GGT GGA ATG AGA CTA TTG
	Reverse	TTC TGA GGC ATC AAC TGA CAG GTC
mIL6	Forward	CAA CCA AGA GGT GAG TGC TTC
	Reverse	GGT GTC CTC TTT CCC ACA CTG
mIsg15	Forward	AGC AAT GGC CTG GGA CCT AAA
	Reverse	AGC CGG CAC ACC AAT CTT
mIfi44	Forward	TGC ACT CTT CTG AGC TGG TG
	Reverse	CCA GCT TGG ACT TCA CAG GA
mCcl5	Forward	GTG CCC ACG TCA AGG AGT AT
	Reverse	CTC TGG GTT GGC ACA CAC TT
mGbp2	Forward	CAC ACC AAG GGC ATC TGG AT
	Reverse	CAC ATA GTG CAG CTG GTC CA
mCcl4	Forward	TGT CTG CCC TCT CTC TCC TC
	Reverse	TAC TCA GTG ACC CAG GGC TC
mIl1β	Forward	CTG CAG CTG GAG AGT GTG G
	Reverse	GGG GAA CTC TGC AGA CTC AA
mIfitm3	Forward	TAT GAG GTG GCT GAG ATG GG
	Reverse	TCA CCC ACC ATC TTC CGA TC
mIrf7	Forward	CCC AGA CTG CCT GTG TAG ACG
	Reverse	CCA GTC TCC AAA CAG CAC TCG
mCxcl10	Forward	GCC GTC ATT TTC TGC CTC A
	Reverse	CGT CCT TGC GAG AGG GAT C
